# Long-term medical imaging use in children with central nervous system tumors

**DOI:** 10.1371/journal.pone.0248643

**Published:** 2021-04-21

**Authors:** Erin J. A. Bowles, Diana L. Miglioretti, Marilyn L. Kwan, Ute Bartels, Adam Furst, Stephanie Y. Cheng, Cindy Lau, Robert T. Greenlee, Sheila Weinmann, Emily C. Marlow, Alanna K. Rahm, Natasha K. Stout, Wes E. Bolch, Mary Kay Theis, Rebecca Smith-Bindman, Jason D. Pole

**Affiliations:** 1 Kaiser Permanente Washington Health Research Institute, Kaiser Permanente Washington, Seattle, Washington, United States of America; 2 Department of Public Health Sciences, University of California, Davis, Davis, California, United States of America; 3 University of California Davis Comprehensive Cancer Center, Davis, California, United States of America; 4 Division of Research, Kaiser Permanente Northern California, Oakland, California, United States of America; 5 The Hospital for Sick Children, Toronto, Ontario, Canada; 6 ICES, Toronto, Ontario, Canada; 7 Marshfield Clinic Research Institute, Marshfield Clinic Health System, Marshfield, Wisconsin, United States of America; 8 Center for Health Research, Kaiser Permanente Northwest, Portland, Oregon, United States of America; 9 Center for Integrated Health Research, Kaiser Permanente Hawaii, Honolulu, Hawaii, United States of America; 10 Center for Health Research, Genomic Medicine Institute, Geisinger, Danville, Pennsylvania, United States of America; 11 Department of Population Medicine, Harvard Medical School and Harvard Pilgrim Health Care Institute, Boston, Massachusetts, United States of America; 12 Department of Biomedical Engineering, University of Florida, Gainesville, Florida, United States of America; 13 Department of Radiology and Biomedical Imaging, Epidemiology and Biostatistics and The Philip R. Lee Institute for Health Policy, University of California, San Francisco, San Francisco, California, United States of America; 14 Centre for Health Services Research, The University of Queensland, Brisbane, Australia; University of Bern, SWITZERLAND

## Abstract

**Background:**

Children with central nervous system (CNS) tumors undergo frequent imaging for diagnosis and follow-up, but few studies have characterized longitudinal imaging patterns. We described medical imaging in children before and after malignant CNS tumor diagnosis.

**Procedure:**

We conducted a retrospective cohort study of children aged 0–20 years diagnosed with CNS tumors between 1996–2016 at six U.S. integrated healthcare systems and Ontario, Canada. We collected computed topography (CT), magnetic resonance imaging (MRI), radiography, ultrasound, nuclear medicine examinations from 12 months before through 10 years after CNS diagnosis censoring six months before death or a subsequent cancer diagnosis, disenrollment from the health system, age 21 years, or December 31, 2016. We calculated imaging rates per child per month stratified by modality, country, diagnosis age, calendar year, time since diagnosis, and tumor grade.

**Results:**

We observed 1,879 children with median four years follow-up post-diagnosis in the U.S. and seven years in Ontario, Canada. During the diagnosis period (±15 days of diagnosis), children averaged 1.10 CTs (95% confidence interval [CI] 1.09–1.13) and 2.14 MRIs (95%CI 2.12–2.16) in the U.S., and 1.67 CTs (95%CI 1.65–1.68) and 1.86 MRIs (95%CI 1.85–1.88) in Ontario. Within one year after diagnosis, 19% of children had ≥5 CTs and 45% had ≥5 MRIs. By nine years after diagnosis, children averaged one MRI and one radiograph per year with little use of other imaging modalities.

**Conclusions:**

MRI and CT are commonly used for CNS tumor diagnosis, whereas MRI is the primary modality used during surveillance of children with CNS tumors.

## Introduction

Central nervous system (CNS) tumors are the second most common pediatric malignancy after leukemia in children under the age of 19 years, accounting for one in four childhood cancers [1–5]. CNS tumor prognosis is variable depending on the type and grade of the tumor. Five-year survival rates for several common tumor types including pilocytic and diffuse astrocytomas range from 80–95%; however, high-grade tumors such as anaplastic astrocytomas or glioblastomas have lower five-year survival rates in the range of 20–25% [2, 5].

In contrast to children with solid tumors [6–10], no clear outcome data support a particular imaging regimen for children with CNS tumors during long-term follow-up. A recent review of studies of children with brain tumors showed that the median time to relapse across all tumor types was 13.7 months [11]. While they did not evaluate imaging surveillance frequency, the authors concluded that routine surveillance may provide no benefit beyond 10 years after diagnosis [11]. The Children’s Oncology Group recommend surveillance imaging starting two years after completing treatment while the National Comprehensive Cancer Network recommendations for surveillance imaging vary depending on the type of tumor, histology, grade, and other factors [12, 13]. The Scottish Intercollegiate Guidelines Network and Dutch Childhood Oncology Group endorse long-term surveillance imaging without offering specific recommendations on modality or frequency [14, 15]. International, harmonized guidelines are under development [16–18]. Because of the relatively high survival rates of these children, concerns about ionizing radiation exposure from repeated computed tomography (CT) have been raised, and studies in pediatric cancer patients have noted a shift in CT use to magnetic resonance imaging (MRI), which does not use ionizing radiation [19, 20].

The purpose of this paper was to describe long-term rates of medical imaging among 1,879 children diagnosed <21 years of age with CNS tumors between 1996–2016 from six U.S. healthcare systems or in Ontario, Canada. We characterized imaging rates from 12 months before cancer diagnosis through 10 years after diagnosis. We included the 12-month period before diagnosis because no previous study has described imaging patterns leading up to diagnosis. We examined imaging trends over time, by patient and tumor characteristics, and by modality to understand imaging practices in a large, North American cohort.

## Materials and methods

The Radiation Induced Cancers (RIC) study is a National Institutes of Health funded retrospective cohort designed to evaluate fetal and childhood imaging exposure and subsequent cancer risk [21, 22]. This analysis included data from six U.S. integrated health care systems (Harvard Pilgrim Health Care, Kaiser Permanente (KP) Hawaii, KP Northern California, KP Northwest, KP Washington, and Marshfield Clinic Health System) and five tertiary care centers in Ontario, Canada, that treat children with cancer (Children’s Hospital of Eastern Ontario, Kingston Health Science Centre, Hospital for Sick Children, McMaster Children’s Hospital and Children’s Hospital, London Health Science Centre). The data included in this analysis are a subset of the RIC study data and include all children diagnosed with CNS tumors from sites with complete tumor data capture. All study sites obtained human subjects approval with a waiver of consent from the following ethics committees or Institutional Review Boards (IRBs): Harvard Pilgrim Health Care IRB, KP Hawaii IRB, KP Northern California IRB, KP Research Compliance Northwest Region, KP Washington Human Subjects Research Committee, Marshfield Clinic Research Institute IRB, and The University of Toronto Health Sciences Ethics Board.

### Population and observation period

We initially included 4,240 children <21 years old diagnosed with a primary CNS tumor documented by local tumor registries between January 1, 1996 through December 31, 2016. Children had to be enrolled in one of the U.S. integrated health systems or eligible for Ontario provincial health coverage through the Ontario Health Insurance Plan (OHIP) for six months before and after diagnosis unless they were diagnosed before six months of age. Children without six months of follow-up after diagnosis (due to death, disenrollment, or a second cancer diagnosis, N = 1,769) or benign CNS tumors (defined when the 5^th^ digit of the morphology code, or the behavior code = 0, N = 592) were excluded. The final sample size included 1,879 children with a primary CNS tumor. Children were observed from up to one year before through 10 years after their CNS tumor diagnosis. Follow-up time was censored at the earliest of disenrollment from the health plan, six months before death or a subsequent cancer diagnosis, 10 years after CNS tumor diagnosis, age 21 years, or end of follow-up (December 31, 2016).

### Cancer registry data and tumor classification

Cancer diagnoses were identified via linkages between the U.S. health plans and tumor registries (either Surveillance Epidemiology and End Results [SEER] or local registries) and in Ontario, Canada, through a linkage with the Ontario Cancer Registry. We included children diagnosed with brain, endocrine, or other CNS tumors initially classified by the International Classification of Childhood Cancers version 3, and then subclassified using International Classification of Diseases for Oncology version 3 codes C700-C729 and C751-C759 [23]. We collected data on age and date of diagnosis, sex, race and ethnicity (U.S. only), cancer site, morphology, grade, and tumor behavior. In addition, we used morphology codes to categorize CNS tumors into specific CNS tumor types using the 2016 World Health Organization (WHO) Classification of Tumors of the CNS [24, 25]. Cancers were graded into four levels according to the 2016 WHO classification of tumors of the central nervous system [24, 25]. “Unknown grade” was assigned to tumors classified as glioma, not otherwise specified (NOS). Tumors with unknown grade were excluded from analyses stratifying by tumor grade.

### Imaging data

Methods to collect and categorize imaging data have been previously described [21, 22]. Briefly, in the U.S., we identified imaging examinations using a combination of electronic medical record and external claims billing data. Examinations were identified using Current Procedural Terminology, International Classification of Disease (ICD)-9, ICD-10, and Healthcare Common Procedure Coding System billing codes [22]. We mapped all billing codes and modifiers over time to an anatomic area (head/brain, chest, neck, cardiac, abdomen [including all examinations of abdomen and/or pelvis], spine, extremity, and unknown/other) and modality (CT, MRI, ultrasound, nuclear medicine, and routine radiographs). We excluded imaging performed in combination with radiation treatment planning because these are often done daily, every time radiation treatment is given, and including them would have inflated imaging rates. To avoid over-counting, we included a maximum of one imaging test per modality and anatomic area per day.

In Ontario, we used Canadian Classification of Health Intervention codes to identify imaging events from the following administrative databases: Discharge Abstract Database (DAD), Same-Day Surgery (SDS), National Ambulatory Reporting System (NACRS) and physician billing codes in OHIP. DAD captures information on all hospitalizations in the province, while the SDS and NACRS captures ambulatory visits including emergency department visits. We used a crosswalk to link Canadian imaging codes to a U.S. counterpart code, allowing analyses to be performed using consistently defined events.

### Statistical approach

Data from the U.S. were pooled across health plans and aggregated into counts by image type and time relative to the “diagnosis period”. Data from Ontario were aggregated similarly. Aggregate data were sent to a central site for analysis. The “diagnosis period” was centered at the diagnosis date and included the 15 days before and after diagnosis (±15 days) to characterize imaging exams associated with the CNS tumor diagnostic process. We calculated monthly imaging rates per child for the 12 months before and after the diagnosis period for each modality (by dividing the number of exams by the number of person-days and multiplying by 30). We calculated yearly imaging rates after diagnosis by summing the monthly data across the entire prior year, dividing by the person-time, and then multiplying the imaging rates by 12. We used an exact Poisson distribution to calculate 95% confidence intervals for all rates.

We calculated imaging rates separately for the U.S. and Ontario and stratified by: 1) age at diagnosis; 2) calendar year of diagnosis; 3) WHO tumor grade; and 4) organ system imaged (head vs. other body part). We evaluated imaging rates by grade, and not tumor type, because grade is designed to be a better predictor of CNS tumor prognosis and severity [24]. We calculated the distribution of the cumulative number of imaging tests per child within the 12 months before and after diagnosis restricting to children with complete follow-up during those time periods. Most analyses were done in SAS (Cary, NC) except confidence intervals were calculated in Stata (College Station, TX).

## Results

In total, 1,879 children diagnosed with CNS tumors between 1996–2016 (475 at U.S. sites and 1,404 in Ontario, Canada, Table 1) were included in this study. The median age at diagnosis was eight years in both the U.S. and Ontario; 55% of children were male. The median follow-up after diagnosis was four years in the U.S. and seven years in Ontario. Nearly 90% of tumors were diagnosed in the brain, 10% in the cranial nerves or other nervous system, and <1% in the endocrine system. The most common tumor types were other astrocytic tumors (32%), diffuse astrocytic and oligodendroglial (21%), embryonal (20%), and glioma NOS (16%). Most tumors were grade I/II (51%) versus grade III/IV (33%).

**Table 1:** Characteristics of children diagnosed with central nervous system tumors between 1996–2016 in the U.S. and Ontario, Canada.

Characteristic	Total		U.S. sites		Ontario	
	N	(%)	N	(%)	N	(%)
**Total N**	1879		475		1404	
**Median follow-up years** [IQR]	n/a		4	[2–7]	7	[3–11]
**Median age** [IQR]	n/a		8	[4–14]	8	[4–13]
**Calendar year of diagnosis**						
1996–2002	585	(31%)	119	(25%)	466	(33%)
2003–2009	633	(34%)	171	(36%)	462	(33%)
2010–2016	661	(35%)	185	(39%)	476	(34%)
**Age at diagnosis, years**						
0–3	452	(24%)	137	(29%)	315	(22%)
4–10	696	(37%)	149	(31%)	547	(39%)
11–15	422	(22%)	103	(22%)	319	(23%)
16–20	309	(16%)	86	(18%)	223	(16%)
**Sex**						
Female	838	(45%)	207	(44%)	631	(45%)
Male	1041	(55%)	268	(56%)	773	(55%)
**Cancer site**						
Brain	1661	(88%)	405	(85%)	1256	(89%)
Cranial Nerves and Other Nervous System	191	(10%)	64	(13%)	127	(9%)
Other Endocrine including Thymus	27	(1%)	6	(1%)	21	(1%)
**WHO classification**						
Choroid plexus	12	(1%)	1	(0%)	11	(1%)
Diffuse astrocytic and oligodendroglial	397	(21%)	98	(21%)	299	(21%)
Embryonal	375	(20%)	85	(18%)	290	(21%)
Ependymal	155	(8%)	32	(7%)	123	(9%)
Glioma NOS	302	(16%)	93	(20%)	209	(15%)
Meningioma	8	(0%)	2	(0%)	6	(0%)
Neuronal and mixed neuronal-glial	16	(1%)	1	(0%)	15	(1%)
Other astrocytic	595	(32%)	159	(33%)	436	(31%)
Tumors of the pituitary and pineal gland	19	(1%)	4	(1%)	15	(1%)
**WHO grade**						
Unknown	302	(16%)	93	(20%)	209	(15%)
Low: I/II	966	(51%)	236	(50%)	730	(52%)
High: III/IV	611	(33%)	146	(31%)	465	(33%)

^a^IQR: inter-quartile range

^b^WHO: World Health Organization

^c^n/a: not applicable

**Fig 1:**
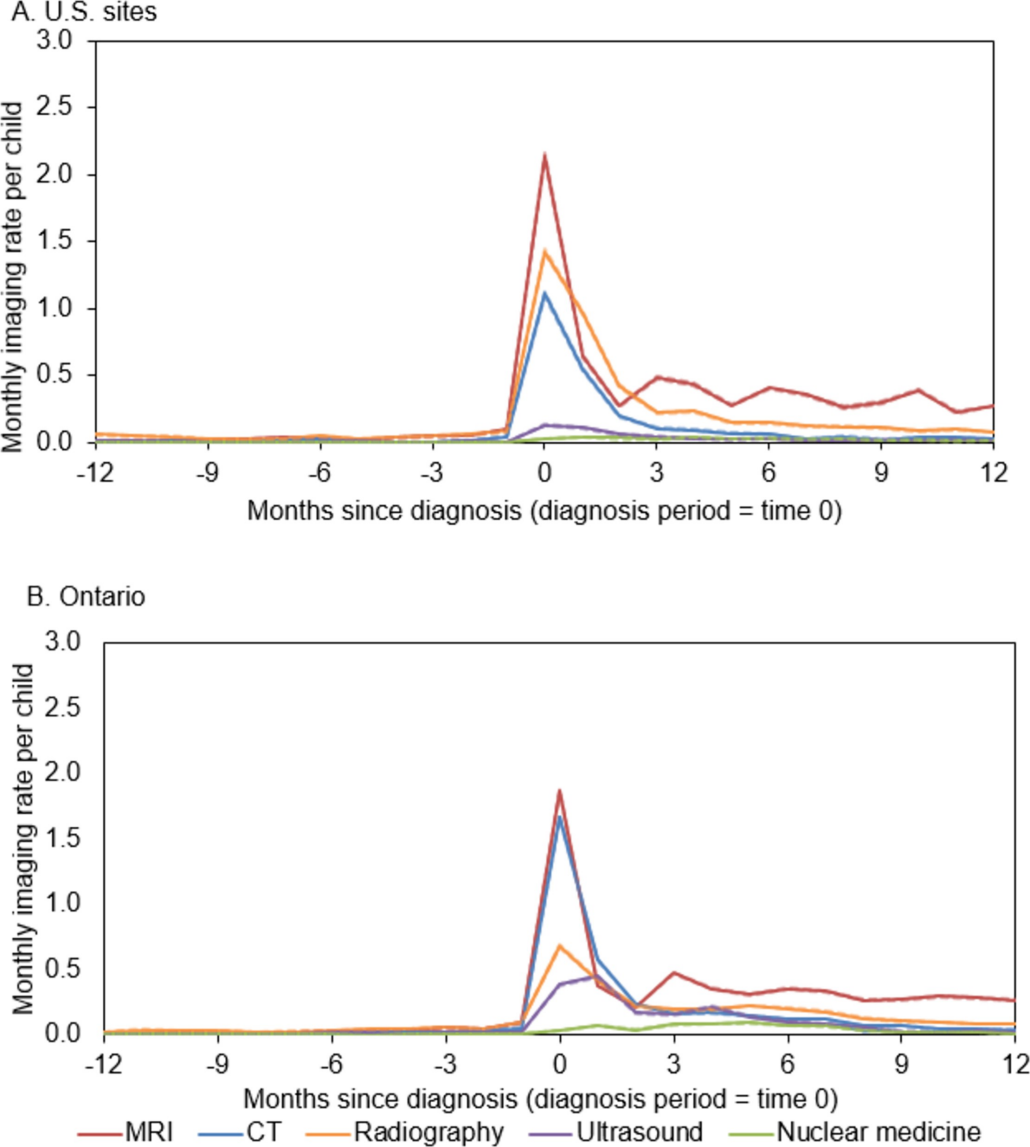
Monthly imaging rates by modality in the 12 months before and after CNS tumor diagnosis in U.S. sites (1A) and Ontario, Canada (1B). This figure shows the monthly imaging rate per child for each imaging modality (MRI [magnetic resonance imaging], CT [computed topography], radiography, ultrasound, and nuclear medicine) in the 12 months before and after the diagnosis period (time 0, or +/-15 days around the day of diagnosis) stratified by U.S. sites (1A) and Ontario, Canada (1B). The 95% confidence intervals are shown by dashed lines and very closely overlap with the imaging rates.

Imaging rates were highest during the diagnosis period (±15 days of diagnosis) in the U.S. (Fig 1A) and Ontario, Canada (Fig 1B). During the diagnosis period, children received an average of 2.13 MRI exams (95%CI 2.12–2.16), 1.42 radiographs (95%CI 1.40–1.44), and 1.11 CT exams (95%CI 1.09–1.13) in the U.S., and 1.86 MRI exams (95%CI 1.85–1.88), 0.68 radiographs (95%CI 0.67–0.69) and 1.67 CT exams (95%CI 1.65–1.68) in Ontario (S1 Table). During the 12 months after the diagnosis period, MRI was the most common exam; rates peaked at three months (0.5 exams per child per month), six months (0.4), and nine months (0.3) in both the U.S. and Ontario, Canada.

**Fig 2:**
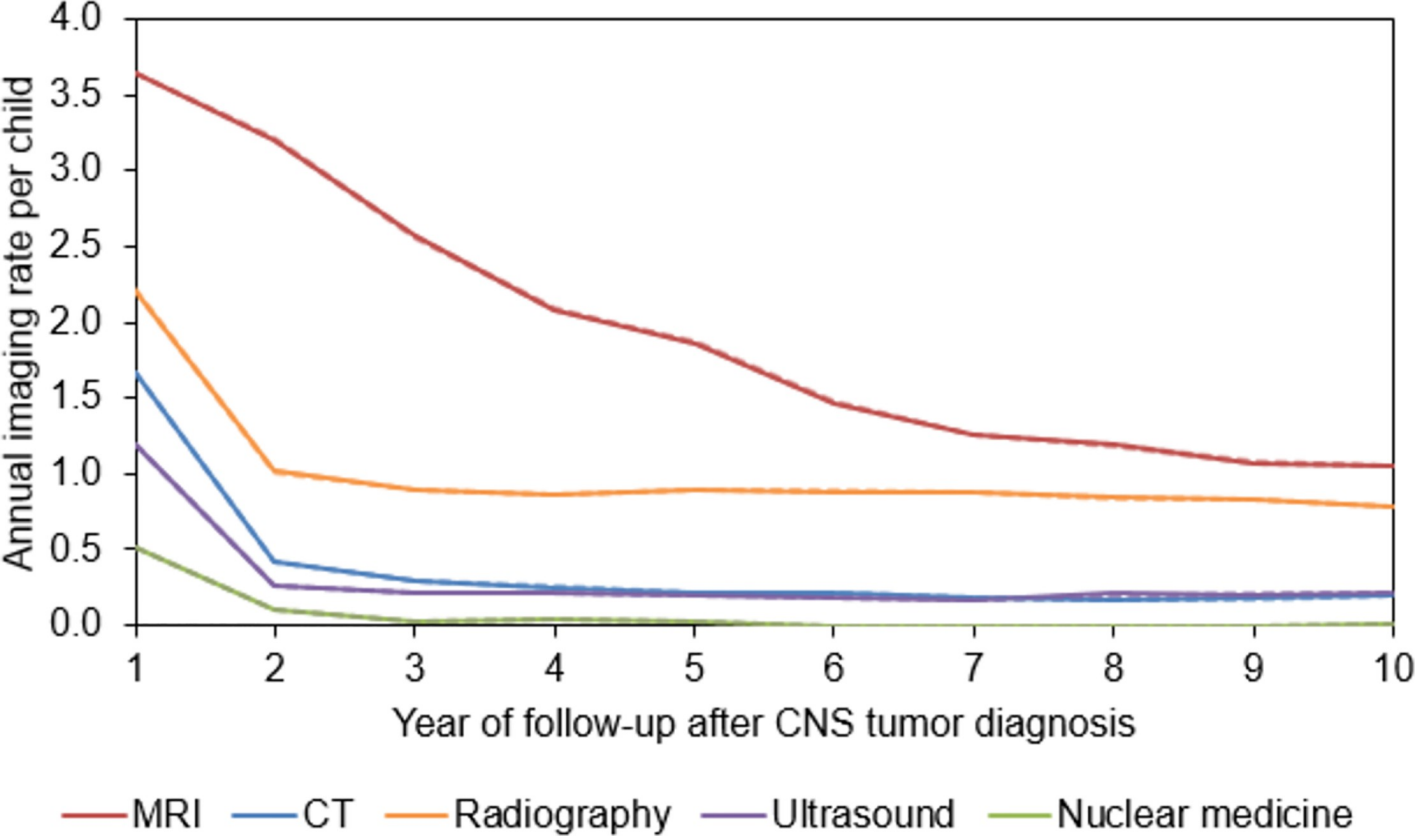
Annual imaging rates by modality and years since CNS tumor diagnosis for U.S. sites and Ontario, Canada combined. This figure shows the annual imaging rate per child for each imaging modality (MRI [magnetic resonance imaging], CT [computed topography], radiography, ultrasound, and nuclear medicine) for up to 10 years after CNS (central nervous system) tumor diagnosis. The 95% confidence intervals are shown by dashed lines and very closely overlap with the imaging rates.

Imaging rates beyond the first year after the diagnosis period were similar between U.S. sites and Ontario, Canada, and are combined in Fig 2. Imaging rates beyond the first year stratified by the U.S. sites and Ontario are shown separately in S1A and S1B Fig. When looking at the combined rates, MRI was the most common imaging exam used for long-term follow-up. Yearly MRI rates declined with follow-up time from 3.64 (95%CI 3.64–3.65) exams per child in the first year after diagnosis to 1.08 (95%CI 1.07–1.08) exams per child beyond nine years of follow-up. Radiography was the next most common surveillance examination, with 2.21 (95%CI 2.21–2.22) exams per child in the first year after the diagnosis period, then dropping to 0.90 (95%CI 0.89–0.90) examinations per year by three years of follow-up. CT examinations occurred at a rate of 1.67 (95%CI 1.66–1.67) per child in the first year after the diagnosis period, then dropped to 0.42 (95%CI 0.41–0.42) per child in the second year of follow-up and continued to decline thereafter. Ultrasound rates followed a pattern very similar to CT. Nuclear medicine was the least common imaging examination during follow-up, and rarely occurred after two years of follow-up.

**Fig 3:**
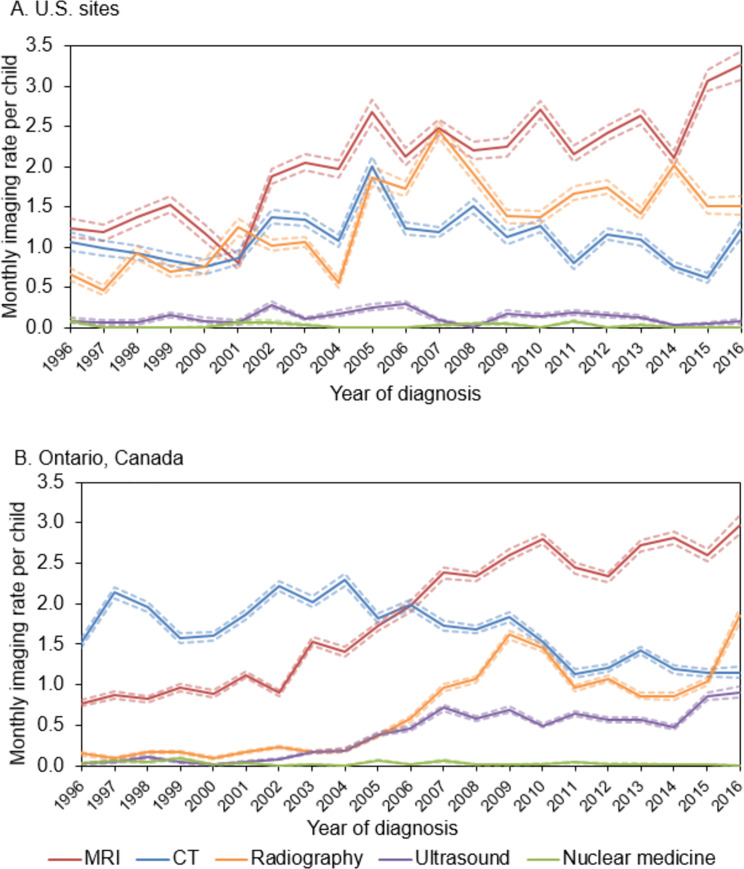
Imaging rates during the CNS tumor diagnosis period over time, stratified by modality for U.S. sites (3A) and Ontario, Canada (3B). This figure shows the monthly imaging rate per child for each imaging modality (MRI [magnetic resonance imaging], CT [computed topography], radiography, ultrasound, and nuclear medicine) during the CNS (central nervous system) tumor diagnosis period (+/-15 days around the day of diagnosis) over years of diagnosis from 1996–2016 stratified by U.S. sites (3A) and Ontario, Canada (3B). The 95% confidence intervals for each modality are shown by dashed lines.

Imaging patterns during the diagnosis period differed by calendar year and between the U.S. and Ontario, Canada (Fig 3). In the U.S., MRI rates were consistently higher than CT rates, and increased steadily over time from 1.22 (95%CI 1.12–1.35) exams per child in 1996 to 3.26 (95%CI 3.08–3.44) in 2016 (Fig 3A). CT rates started out low in the U.S., peaked at 2.00 (95%CI 1.87–2.13) exams per child in 2005, then dropped down to a low of 0.61 (95%CI 0.55–0.68) exams per child in 2015. Radiography rates surpassed CT rates in 2006 and tended to be higher in the U.S. compared with Ontario.

During the diagnosis period in Ontario, CT rates started out higher than MRI, around 2 exams per child until 2006, then declined to 1.15 (95%CI 1.08–1.23) exams in 2016 (Fig 3B). MRI rates steadily increased over time surpassing CT rates in 2006 to a high of 2.97 (95%CI 2.86–3.09) exams per child in 2016. Ultrasound and nuclear medicine imaging rates were low across time in both countries, though ultrasound rates did increase in Ontario to 0.46 (95%CI 0.43–0.49) exams per child in 2006 and 0.91 (95%CI 0.85–0.98) exams per child in 2016.

**Fig 4:**
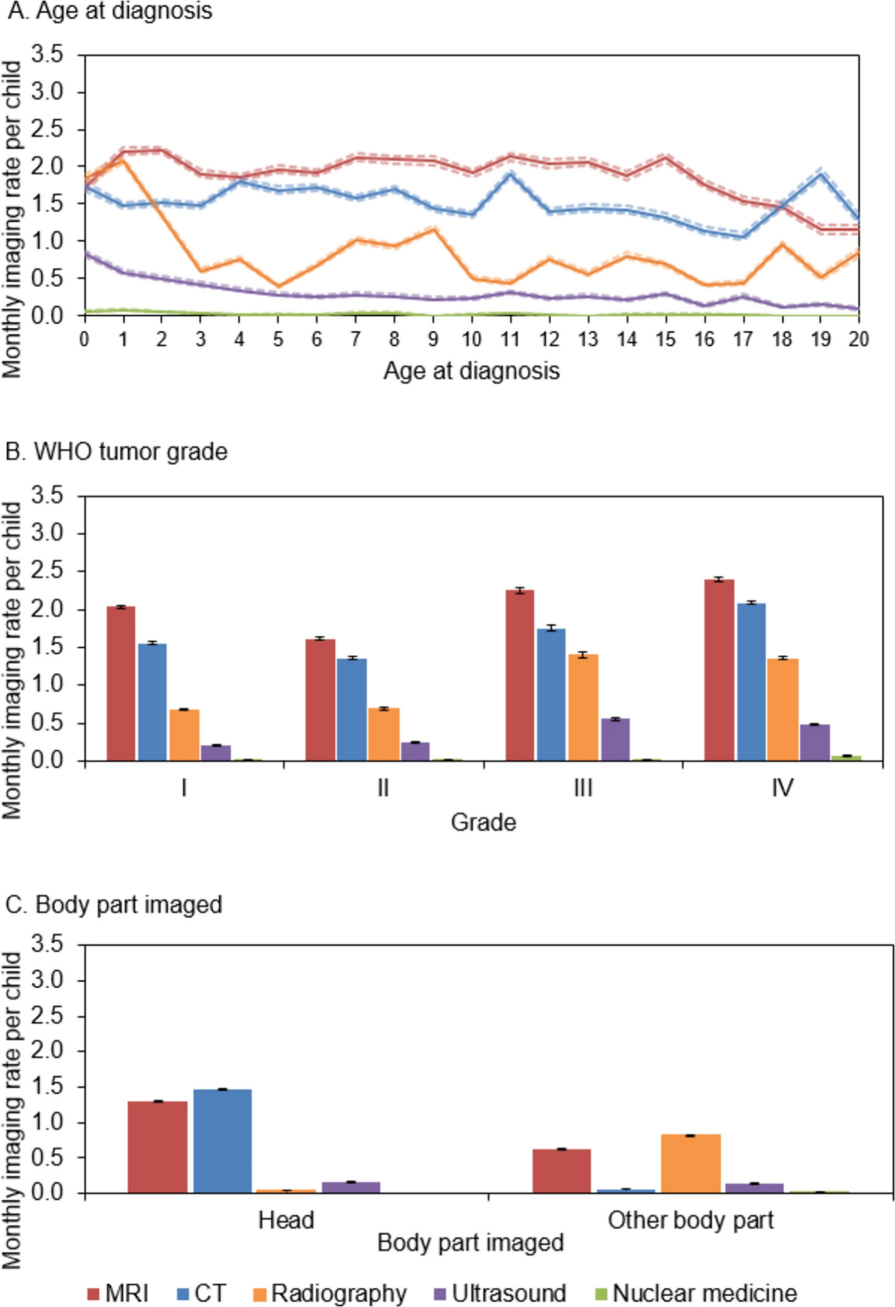
MRI and CT imaging rates during the CNS tumor diagnosis period in U.S. sites and Ontario, Canada combined by child’s age at diagnosis (4A), tumor grade (4B), and body part imaged (4C). This figure shows the monthly imaging rate per child for each imaging modality (MRI [magnetic resonance imaging], CT [computed topography], radiography, ultrasound, and nuclear medicine) during the CNS (central nervous system) tumor diagnosis period (+/-15 days around the day of diagnosis) stratified by age at diagnosis (4A), tumor grade (4B), and body part imaged (4C, stratified by head vs. other). The 95% confidence intervals for each modality are shown by dashed lines in 4A and error bars in 4B and 4C.

Imaging patterns during the diagnosis period differed by age at diagnosis, tumor grade, and body part imaged but were similar between the U.S. and Ontario. Imaging rates for age and grade stratified by the U.S. sites and Ontario are shown separately in S2A and S2B, S3A and S3B and S4A and S4B Figs. MRI was the most common imaging modality among children age <18 at the time of diagnosis (Fig 4A). MRI rates at diagnosis remained stable around 2 exams per child, until age 15, then dropped to a low of 1.16 (95%CI 1.10–1.22) MRI examinations per child at age 20 years. CT rates hovered around 1.5 examinations per child during the diagnosis period with small increases in imaging at ages 11 and 19 years. Radiography and ultrasound were more common among children ages 0–1 years compared to older children, though MRI and CT were still more common than ultrasound in this age group.

MRI rates were higher than CT for each tumor grade category, and highest for grade IV at 2.40 (95%CI 2.37–2.42) MRI examinations per child during the diagnosis period (Fig 4B). CT rates were also highest for grade IV tumors at 2.09 (95%CI 2.06–2.11) examinations per child during the diagnosis period. Children with CNS tumors had an average of 1.30 (95%CI 1.29–1.31) MRIs on the head and 0.63 (95%CI 0.63–0.64) MRIs on other parts of the body during the diagnosis period (Fig 4C). CT exams of the head were also common during the diagnosis period at a rate of 1.47 exams per child (95% 1.46–1.48). Radiographs were the most common exam of other body parts during the diagnosis period at 0.81 exams per child (95%CI 0.81–0.82).

More children had multiple CT and MRI examinations in the 12 months after diagnosis than the 12 months before (S5A and S5B Fig). In the 12 months before the diagnosis period, a small number of children (<1%) had ≥5 CT examinations with a maximum of 21 CTs during this period. During the same time period, the number of MRI exams per children ranged from 0–13, with 2% of children having ≥5. In the 12 months after the diagnosis period, the number of CTs per child ranged from 0–30 with 19% of children having ≥5 CT examinations. During the same time period, the number of MRIs per child ranged from 0–21; 45% of children had ≥5 MRIs in the 12 months after the diagnosis period.

## Discussion

Among 1,879 children with CNS tumors from the U.S. and Ontario, Canada, MRI use was more common than other imaging modalities, particularly during long-term follow-up and in recent calendar years. The exception to this was during the diagnosis period: children had an average of 1 CT exam and 2 MRI exams in the U.S., and almost 2 CT exams and 2 MRI exams per child in Ontario, Canada. While CT exams are often used initially to rapidly diagnose children with new neurologic concerns, MRI often follows to better define a mass seen on CT imaging, for staging, to look for spinal metastases, or to help with neurosurgical planning [19, 26]. After diagnosis, MRI rates averaged 1 MRI per child per year for up to 10 years. In contrast, CT imaging was used infrequently beyond two years after diagnosis.

This study describes long-term imaging patterns using electronic healthcare data in children with CNS tumors both leading up to diagnosis and during long-term follow-up. We noted a decline in CT use during the diagnosis period during the mid-2000’s. The decline in CT use in our study may be related to increased awareness of stochastic cancer risks from ionizing radiation exposure (specifically from CT imaging) and the “Image Gently” campaign, which started in 2007 [27]. We noted a corresponding increase in MRI use over time among children with CNS tumors, suggesting that MRI is replacing CT use for imaging during follow-up. This may be reassuring to patients and their families because, unlike CT, MRI does not expose children to ionizing radiation. Ionizing radiation exposure has the potential to increase the risk for subsequent cancers [10, 19].

The only other common type of imaging used during the diagnosis period was radiography, which surpassed CT rates in 2006 and tended to be higher in the U.S. compared with Ontario. A possible explanation may be due to differences in the use of programmable shunts between the U.S. and Ontario. Programmable shuts require head radiographs after MRI to double-check and/or readjust programmed pressure; however, this difference in utilization has not been evaluated to the best of our knowledge. In addition, radiography exams on other parts of the body (other than the head) were more common in the U.S. sites compared to Ontario during the diagnosis period. Radiography and ultrasound were more common during the diagnosis period in children <1 year of age than older children, likely because of the complexity involved in using other imaging modalities in very young children.

We noted small peaks in MRI use at 3, 6, 9, and 12 months after the diagnosis period (and likely during active treatment) which correspond to standard clinical practice in both the U.S. and Canada. MRI use continued through 10 years post-diagnosis with approximately 1 examination per child per year. Imaging rates beyond 12 months follow-up were consistent between the U.S., Ontario, Canada, and current recommendations for periodic evaluations that include imaging from the Children’s Oncology and long-term surveillance imaging from the National Comprehensive Cancer Network [12, 13].

There has been a call for more collaborative, large observational studies on descriptions of surveillance after childhood cancer diagnoses, adherence to surveillance guidelines, and evaluation of long-term outcomes [17, 28]. In addition, the National Cancer Institute is investing in childhood cancer research by promoting data generation and sharing, infrastructure development, and interventions in childhood cancer survivors <21 years of age [29–31]. Our study only describes imaging patterns; however, in describing imaging, it is important to consider the balance of the benefits and harms of imaging. The potential benefits of conducting frequent and long-term surveillance at rates sufficient to detect early progression of disease, recurrence, and secondary malignancy–or give the patient reassurance that these outcomes have not developed—must be balanced with the potential risks from over-imaging. Potential long-term harms and unknown risks from MRI include detrimental cognitive effects from repeated sedation [32–34] (although reported results have been inconsistent) and unknown effects of retaining gadolinium-based contrast agents in the body [35]. In addition, MRI may not be appropriate for all children or all types of tumors (for example, children who have internal surgical clips that could lead to artifacts interfering with interpretation, children unable to remain still or with contraindications for sedation, or children with claustrophobia or intolerance for noise) and of limited diagnostic value in some CNS tumors [19]. As follow-up time increases, it will be increasingly important to understand the best practices for childhood cancer survivors [8]. Regardless of the imaging modality, patients and families may experience anxiety associated with waiting for results of a surveillance imaging test. The implementation of standard guidelines for imaging in childhood cancer survivors will greatly improve the quality of follow-up care, including detection of second malignancies, while reducing potential harms from ionizing radiation, costs, and anxiety [4, 17, 18].

Our study has several limitations. The indication for imaging was not available in our study data, so we could not distinguish between imaging tests performed because of patient-reported symptoms of disease versus those acquired for surveillance purposes. Therefore, we did not include imaging that occurred within six months before a subsequent cancer diagnosis or death to reduce the chances of including imaging performed for signs or symptoms of a recurrence or second cancer. We also did not know which imaging modalities used anesthesia or sedation in our study data, which might have influenced the patient’s choice of modality and perceived risk-benefit. We were not able to account for tumor progression, which is not routinely collected in tumor registries. We were not able to evaluate imaging rates stratified by treatment received, which may be critical in determining appropriate surveillance recommendations. Our confidence intervals may be slightly underestimated because some children may have multiple exams within a 30-day period. Finally, this study included children enrolled in U.S. integrated healthcare systems, and imaging patterns in our U.S. population may be different than imaging patterns in children covered by fee-for-service plans or children without health insurance. If imaging was done outside the health care system and billed to a different insurer, this would not have been captured in our analysis. Imaging patterns in Canada were assessed only among children in the province of Ontario. However, it is reassuring that imaging patterns were similar between the two countries, suggesting that they may be generalizable to other parts of the U.S. and Canada.

Our study also has several strengths. Our study comprises one of the largest populations of children with CNS tumors in North America. We used automated data to ascertain imaging records so that data collection was not biased by self-report. All children included in our study had health insurance, greatly reducing the likelihood that we were missing any claims for imaging codes in automated data. We had post-diagnosis follow-up data for a median of four years at U.S. sites and seven years among Ontario children, and up to 10 years on some children, enabling us to report on long-term imaging use on a population-level. Finally, the geographic distribution of our study sites across North America further increases the potential generalizability of our results to other regions of the U.S. and Canada.

## Conclusions

Imaging tests are one of the best tools to diagnose solid cancers in children and monitor for treatment effectiveness, cancer progression, and long-term sequelae. Our study describes patterns of imaging tests leading up to diagnosis and for 10 years of follow-up in children with CNS tumors. CT and MRI were both used to diagnose CNS tumors in children. In the first year after diagnosis, children received multiple imaging exams–usually MRI—every three months. After the first year, MRI was the most common surveillance imaging examination occurring approximately once per year for up to 10 years after diagnosis. Future research should be directed toward the evaluation of long-term benefits of annual MRI examinations in children, and whether any potential harms outweigh the benefits.

## Supporting information

S1 Table Monthly imaging rates in children diagnosed with brain or central nervous system tumors before and after diagnosis. This table shows the detailed monthly imaging rates by modality before and after diagnosis, stratified by U.S. sites and Ontario, Canada.
(DOCX)

S1 Fig Annual imaging rates by modality and years since CNS diagnosis stratified by U.S. sites (S1A) and Ontario, Canada (S1B). This figure shows the annual imaging rate per child for each imaging modality (MRI [magnetic resonance imaging], CT [computed topography], radiography, ultrasound, and nuclear medicine) for up to 10 years after CNS (central nervous system) tumor diagnosis stratified by U.S. sites (S1A) and Ontario (S1B). The 95% confidence intervals are shown by dashed lines and very closely overlap with the imaging rates.
(TIFF)

S2 Fig MRI and CT imaging rates during the CNS diagnosis period by child’s age at diagnosis stratified by U.S. sites (2A) and Ontario, Canada (2B). This figure shows the monthly imaging rate per child for each imaging modality (MRI [magnetic resonance imaging], CT [computed topography], radiography, ultrasound, and nuclear medicine) during the CNS (central nervous system) tumor diagnosis period (+/-15 days around the day of diagnosis) stratified by age at diagnosis and by U.S. sites (S2A) and Ontario (S2B). The 95% confidence intervals are shown by dashed lines and very closely overlap with the imaging rates.
(TIFF)

S3 Fig MRI and CT imaging rates during the CNS diagnosis period by CNS tumor grade stratified by U.S. sites (3A) and Ontario, Canada (3B). This figure shows the monthly imaging rate per child for each imaging modality (MRI [magnetic resonance imaging], CT [computed topography], radiography, ultrasound, and nuclear medicine) during the CNS (central nervous system) tumor diagnosis period (+/-15 days around the day of diagnosis) stratified by CNS tumor grade and by U.S. sites (S3A) and Ontario (S3B). The 95% confidence intervals are shown by error bars.
(TIFF)

S4 Fig MRI and CT imaging rates during the CNS diagnosis period by body part imaged by U.S. sites (4A) and Ontario, Canada (4B). This figure shows the monthly imaging rate per child for each imaging modality (MRI [magnetic resonance imaging], CT [computed topography], radiography, ultrasound, and nuclear medicine) during the CNS (central nervous system) tumor diagnosis period (+/-15 days around the day of diagnosis) stratified by body part imaged and by U.S. sites (S4A) and Ontario (S4B). The 95% confidence intervals are shown by error bars.
(TIFF)

S5 Fig Distribution of number of computed tomography (CT) and magnetic resonance imaging (MRI) exams among children with CNS tumors in the 12 months before (S5A) and after (S5B) diagnosis in U.S. sites and Ontario, Canada, combined. This figure shows the proportion of children by the number of CT (computed topography) and MRI (magnetic resonance imaging) exams they had in the 12 months before CNS (central nervous system) tumor diagnosis (5A) and the 12 months after CNS diagnosis (5B).
(TIFF)

## References

[1] National Brain Tumor Society [cited 2019 December 4]. Available from: www.braintumor.org.

[2] American Cancer Society [cited 2019 December 4]. Available from: www.cancer.org.

[3] Johnson KJ, Cullen J, Barnholtz-Sloan JS, Ostrom QT, Langer CE, Turner MC, et al. Childhood brain tumor epidemiology: a brain tumor epidemiology consortium review. Cancer Epidemiol Biomarkers Prev. 2014;23(12):2716–36. 10.1158/1055-9965.EPI-14-0207 25192704 PMC4257885

[4] Mariotto AB, Rowland JH, Yabroff KR, Scoppa S, Hachey M, Ries L, et al. Long-term survivors of childhood cancers in the United States. Cancer Epidemiol Biomarkers Prev. 2009;18(4):1033–40. 10.1158/1055-9965.EPI-08-0988 19336557

[5] Ostrom QT, Gittleman H, Truitt G, Boscia A, Kruchko C, Barnholtz-Sloan JS. CBTRUS Statistical Report: Primary Brain and Other Central Nervous System Tumors Diagnosed in the United States in 2011–2015. Neuro Oncol. 2018;20(suppl_4):iv1–iv86. 10.1093/neuonc/noy131 30445539 PMC6129949

[6] Chavhan GB, Babyn PS, Nathan PC, Kaste SC. Imaging of acute and subacute toxicities of cancer therapy in children. Pediatr Radiol. 2016;46(1):9–20; quiz 6–8. 10.1007/s00247-015-3454-1 26459011

[7] Kaste SC. Oncological imaging: tumor surveillance in children. Pediatr Radiol. 2011;41 Suppl 2:505–8. 10.1007/s00247-011-2108-1 21847730 PMC4700923

[8] Langer T, Grabow D, Steinmann D, Wormann B, Calaminus G. Late Effects and Long-Term Follow-Up after Cancer in Childhood. Oncol Res Treat. 2017;40(12):746–50. 10.1159/000484936 29183026

[9] Nathan PC, Patel SK, Dilley K, Goldsby R, Harvey J, Jacobsen C, et al. Guidelines for identification of, advocacy for, and intervention in neurocognitive problems in survivors of childhood cancer: a report from the Children’s Oncology Group. Arch Pediatr Adolesc Med. 2007;161(8):798–806. 10.1001/archpedi.161.8.798 17679663

[10] Weiser DA, Kaste SC, Siegel MJ, Adamson PC. Imaging in childhood cancer: a Society for Pediatric Radiology and Children’s Oncology Group Joint Task Force report. Pediatr Blood Cancer. 2013;60(8):1253–60. 10.1002/pbc.24533 23572212 PMC4636336

[11] Otth M, Scheinemann K. Surveillance imaging for high-grade childhood brain tumors: What to do 10 years after completion of treatment? Pediatr Blood Cancer. 2018;65(11):e27311. 10.1002/pbc.27311 30009501

[12] Children’s Oncology Group: Long-term follow-up guidelines for survivors of childhood, adolescent, and young adult cancers [cited 2019 December 4]. Available from: http://survivorshipguidelines.org/pdf/2018/COG_LTFU_Guidelines_v5.pdf. 10.1001/jamaoncol.2024.6812 PMC12188901 39976936

[13] National Comprehensive Cancer Network [Available from: https://www.nccn.org/.

[14] Scottish Intercollegiate Guidelines Network 132—Long term follow up of survivors of childhood cancer [cited 2019 December 4]. Available from: https://www.sign.ac.uk/assets/sign132.pdf.

[15] Guidelines from the Dutch Childhood Oncology Group (DCOG) / SKION—Guidelines for follow-up in survivors of childhood cancer 5 years after diagnosis [cited 2019 December 4]. Available from: https://www.skion.nl/workspace/uploads/vertaling-richtlijn-LATER-versie-final-okt-2014_2.pdf.

[16] International Guideline Harmonization Group [cited 2019 December 4]. Available from: http://www.ighg.org/guidelines/topics/central-nervous-system-malignancies/.

[17] Bhatia S, Armenian SH, Armstrong GT, van Dulmen-den Broeder E, Hawkins MM, Kremer LC, et al. Collaborative Research in Childhood Cancer Survivorship: The Current Landscape. J Clin Oncol. 2015;33(27):3055–64. 10.1200/JCO.2014.59.8052 26304891 PMC4567704

[18] Kremer LC, Mulder RL, Oeffinger KC, Bhatia S, Landier W, Levitt G, et al. A worldwide collaboration to harmonize guidelines for the long-term follow-up of childhood and young adult cancer survivors: a report from the International Late Effects of Childhood Cancer Guideline Harmonization Group. Pediatr Blood Cancer. 2013;60(4):543–9. 10.1002/pbc.24445 23281199 PMC3819170

[19] Voss SD. Staging and following common pediatric malignancies: MRI versus CT versus functional imaging. Pediatr Radiol. 2018;48(9):1324–36. 10.1007/s00247-018-4162-4 30078040

[20] Morel B, Jaudeau-Collart AC, Proisy M, Leiber LM, Tissot V, Quere MP, et al. Variability in Imaging Practices and Comparative Cumulative Effective Dose for Neuroblastoma and Nephroblastoma Patients at 6 Pediatric Oncology Centers. J Pediatr Hematol Oncol. 2018;40(1):36–42. 10.1097/MPH.0000000000000915 28697171

[21] Kwan ML, Miglioretti DL, Marlow EC, Aiello Bowles EJ, Weinmann S, Cheng SY, et al. Trends in Medical Imaging During Pregnancy in the United States and Ontario, Canada, 1996 to 2016. JAMA Netw Open. 2019;2(7):e197249. 10.1001/jamanetworkopen.2019.7249 31339541 PMC6659354

[22] Smith-Bindman R, Kwan ML, Marlow EC, Theis MK, Bolch W, Cheng SY, et al. Trends in Use of Medical Imaging in US Health Care Systems and in Ontario, Canada, 2000–2016. JAMA. 2019;322(9):843–56. 10.1001/jama.2019.11456 31479136 PMC6724186

[23] International Classification of Childhood Cancer, version 3 [cited 2019 December 4]. Available from: https://seer.cancer.gov/iccc/iccc3.html.

[24] Louis DN, Perry A, Reifenberger G, von Deimling A, Figarella-Branger D, Cavenee WK, et al. The 2016 World Health Organization Classification of Tumors of the Central Nervous System: a summary. Acta Neuropathol. 2016;131(6):803–20. 10.1007/s00401-016-1545-1 27157931

[25] WHO Classification of Tumours of the Central Nervous System. Revised 4th Edition ed2016.

[26] Chu WC, Lee V, Howard RG, Roebuck DJ, Chik KW, Li CK. Imaging findings of paediatric oncology patients presenting with acute neurological symptoms. Clin Radiol. 2003;58(8):589–603. 10.1016/s0009-9260(03)00129-6 12887951

[27] The Image Gently Alliance [cited 2019 December 4]. Available from: https://www.imagegently.org/.

[28] Alfano CM, Smith T, de Moor JS, Glasgow RE, Khoury MJ, Hawkins NA, et al. An action plan for translating cancer survivorship research into care. J Natl Cancer Inst. 2014;106(11). 10.1093/jnci/dju287 25249551 PMC4184343

[29] Jacobsen PB, Rowland JH, Paskett ED, Van Leeuwen F, Moskowitz C, Katta S, et al. Identification of Key Gaps in Cancer Survivorship Research: Findings From the American Society of Clinical Oncology Survey. J Oncol Pract. 2016;12(3):190–3. 10.1200/JOP.2015.009258 26907451

[30] Robison LL, Armstrong GT, Boice JD, Chow EJ, Davies SM, Donaldson SS, et al. The Childhood Cancer Survivor Study: a National Cancer Institute-supported resource for outcome and intervention research. J Clin Oncol. 2009;27(14):2308–18. 10.1200/JCO.2009.22.3339 19364948 PMC2677920

[31] National Cancer Institute Childhood Cancer Data Initiative [cited 2019 December 4]. Available from: https://www.cancer.gov/research/areas/childhood/childhood-cancer-data-initiative.

[32] Chemaly M, El-Rajab MA, Ziade FM, Naja ZM. Effect of one anesthetic exposure on long-term behavioral changes in children. J Clin Anesth. 2014;26(7):551–6. 10.1016/j.jclinane.2014.03.013 25439418

[33] Hu D, Flick RP, Zaccariello MJ, Colligan RC, Katusic SK, Schroeder DR, et al. Association between Exposure of Young Children to Procedures Requiring General Anesthesia and Learning and Behavioral Outcomes in a Population-based Birth Cohort. Anesthesiology. 2017;127(2):227–40. 10.1097/ALN.0000000000001735 28609302 PMC5515677

[34] Ing CH, DiMaggio CJ, Whitehouse AJ, Hegarty MK, Sun M, von Ungern-Sternberg BS, et al. Neurodevelopmental outcomes after initial childhood anesthetic exposure between ages 3 and 10 years. J Neurosurg Anesthesiol. 2014;26(4):377–86. 10.1097/ANA.0000000000000121 25144506

[35] U.S. Food & Drug Administration Safety Communication: FDA warns that gadolinium-based contrast agents (GBCAs) are retained in the body; requires new class of warnings [cited 2020 April 15]. Available from: https://www.fda.gov/drugs/drug-safety-and-availability/fda-drug-safety-communication-fda-warns-gadolinium-based-contrast-agents-gbcas-are-retained-body.

