# Supplementary material for: Long-term medical imaging use in children with central nervous system tumors
Source: PLoS One. 2021 Apr 21;16(4):e0248643. doi: 10.1371/journal.pone.0248643 (PMC8059842; doi:10.1371/journal.pone.0248643)
Supplement: S1 Table — This table shows the detailed monthly imaging rates by modality before and after diagnosis, stratified by U.S. sites and Ontario, Canada. (DOCX) [file pone.0248643.s001.docx]

**S1 Table. Monthly imaging rates in children diagnosed with brain or central nervous system tumors before and after diagnosis.**

| Months since diagnosis (0) | Person days of follow-up | | CT rate per child per month | | | MRI rate per child per month | | | Nuclear medicine rate per child per month | | | Ultrasound rate per child per month | | | Radiography rate per child per month | |
| --- | --- | --- | --- | --- | --- | --- | --- | --- | --- | --- | --- | --- | --- | --- | --- | --- |
|  | U.S. | Ontario | U.S. | Ontario | U.S. | | Ontario | U.S. | | Ontario | U.S. | | Ontario | U.S. | | Ontario |
| -12 | 6,333 | 21,097 | 0.005 | 0.007 | 0 | | 0.010 | 0 | | 0.003 | 0.009 | | 0.004 | 0.057 | | 0.018 |
| -11 | 12,699 | 41,592 | 0.002 | 0.006 | 0.007 | | 0.006 | 0.002 | | 0.001 | 0.009 | | 0.005 | 0.047 | | 0.037 |
| -10 | 13,068 | 41,723 | 0.011 | 0.003 | 0.011 | | 0.019 | 0 | | 0.001 | 0.007 | | 0.006 | 0.041 | | 0.031 |
| -9 | 13,418 | 41,888 | 0.002 | 0.004 | 0.011 | | 0.014 | 0 | | 0.001 | 0.004 | | 0.009 | 0.027 | | 0.036 |
| -8 | 13,643 | 42,025 | 0 | 0.004 | 0.024 | | 0.009 | 0.002 | | 0.001 | 0.000 | | 0.003 | 0.020 | | 0.024 |
| -7 | 13,888 | 42,157 | 0.017 | 0.010 | 0.035 | | 0.020 | 0 | | 0.002 | 0.004 | | 0.006 | 0.024 | | 0.026 |
| -6 | 14,095 | 42,215 | 0.021 | 0.005 | 0.028 | | 0.025 | 0 | | 0.001 | 0 | | 0.015 | 0.045 | | 0.033 |
| -5 | 14,229 | 42,324 | 0.006 | 0.022 | 0.013 | | 0.034 | 0 | | 0 | 0.011 | | 0.013 | 0.027 | | 0.040 |
| -4 | 14,265 | 42,369 | 0.002 | 0.015 | 0.034 | | 0.030 | 0 | | 0.001 | 0.004 | | 0.010 | 0.036 | | 0.046 |
| -3 | 14,344 | 42,504 | 0.002 | 0.014 | 0.046 | | 0.052 | 0.002 | | 0.001 | 0.004 | | 0.022 | 0.046 | | 0.054 |
| -2 | 14,356 | 42,500 | 0.017 | 0.030 | 0.050 | | 0.049 | 0.004 | | 0.001 | 0.010 | | 0.014 | 0.059 | | 0.044 |
| -1 | 14,465 | 42,574 | 0.044 | 0.051 | 0.097 | | 0.091 | 0.002 | | 0.004 | 0.004 | | 0.024 | 0.085 | | 0.095 |
| 0 | 14,456 | 42,776 | 1.108 | 1.667 | 2.140 | | 1.864 | 0.023 | | 0.030 | 0.129 | | 0.382 | 1.424 | | 0.678 |
| 1 | 14,278 | 42,701 | 0.546 | 0.574 | 0.639 | | 0.374 | 0.038 | | 0.068 | 0.109 | | 0.448 | 0.962 | | 0.415 |
| 2 | 14,096 | 42,708 | 0.194 | 0.234 | 0.270 | | 0.208 | 0.038 | | 0.037 | 0.057 | | 0.169 | 0.419 | | 0.217 |
| 3 | 13,869 | 42,697 | 0.106 | 0.162 | 0.480 | | 0.471 | 0.022 | | 0.079 | 0.041 | | 0.154 | 0.218 | | 0.191 |
| 4 | 13,659 | 42,704 | 0.092 | 0.167 | 0.430 | | 0.351 | 0.037 | | 0.084 | 0.024 | | 0.214 | 0.231 | | 0.192 |
| 5 | 13,551 | 42,762 | 0.069 | 0.148 | 0.277 | | 0.307 | 0.024 | | 0.091 | 0.020 | | 0.137 | 0.146 | | 0.220 |
| 6 | 13,366 | 42,567 | 0.061 | 0.123 | 0.404 | | 0.354 | 0.029 | | 0.071 | 0.025 | | 0.101 | 0.144 | | 0.199 |
| 7 | 13,048 | 41,784 | 0.023 | 0.118 | 0.354 | | 0.333 | 0.021 | | 0.065 | 0.011 | | 0.084 | 0.120 | | 0.177 |
| 8 | 12,763 | 40,963 | 0.042 | 0.076 | 0.263 | | 0.262 | 0.024 | | 0.034 | 0.009 | | 0.053 | 0.113 | | 0.124 |
| 9 | 12,487 | 40,364 | 0.019 | 0.071 | 0.296 | | 0.268 | 0.017 | | 0.022 | 0.019 | | 0.025 | 0.108 | | 0.103 |
| 10 | 12,141 | 39,730 | 0.032 | 0.044 | 0.381 | | 0.293 | 0.017 | | 0.016 | 0.010 | | 0.017 | 0.086 | | 0.097 |
| 11 | 11,929 | 38,898 | 0.035 | 0.049 | 0.221 | | 0.281 | 0.008 | | 0.017 | 0.018 | | 0.032 | 0.098 | | 0.083 |
| 12 | 126,320 | 433,223 | 0.023 | 0.038 | 0.272 | | 0.266 | 0.010 | | 0.008 | 0.014 | | 0.024 | 0.078 | | 0.086 |
| 24 | 101,243 | 383,112 | 0.019 | 0.026 | 0.204 | | 0.216 | 0.003 | | 0.002 | 0.009 | | 0.020 | 0.074 | | 0.075 |
| 36 | 79,898 | 339,329 | 0.017 | 0.022 | 0.167 | | 0.175 | 0.005 | | 0.003 | 0.009 | | 0.020 | 0.075 | | 0.071 |

| Months since diagnosis (0) | Person days of follow-up | | CT rate per child per month | | | MRI rate per child per month | | | NUCS rate per child per month | | | Ultrasound rate per child per month | | | Radiography rate per child per month | |
| --- | --- | --- | --- | --- | --- | --- | --- | --- | --- | --- | --- | --- | --- | --- | --- | --- |
|  | U.S. | Ontario | U.S. | Ontario | U.S. | | Ontario | U.S. | | Ontario | U.S. | | Ontario | U.S. | | Ontario |
| 48 | 64,333 | 296,850 | 0.015 | 0.019 | 0.143 | | 0.158 | 0.003 | | 0.002 | 0.009 | | 0.018 | 0.064 | | 0.077 |
| 60 | 49,238 | 261,729 | 0.006 | 0.020 | 0.101 | | 0.126 | 0 | | 0.002 | 0.005 | | 0.017 | 0.069 | | 0.075 |
| 72 | 37,592 | 227,005 | 0.012 | 0.015 | 0.105 | | 0.105 | 0 | | 0.002 | 0.011 | | 0.015 | 0.054 | | 0.076 |
| 84 | 30,436 | 194,278 | 0.013 | 0.015 | 0.096 | | 0.100 | 0 | | 0.001 | 0.006 | | 0.019 | 0.059 | | 0.072 |
| 96 | 26,082 | 164,406 | 0.009 | 0.016 | 0.081 | | 0.091 | 0 | | 0.003 | 0.006 | | 0.019 | 0.044 | | 0.073 |
| 108 | 20,223 | 136,000 | 0.013 | 0.017 | 0.079 | | 0.089 | 0.001 | | 0.001 | 0.016 | | 0.018 | 0.047 | | 0.068 |

S1 Table continued

^a^CT: Computed topography; ^b^MRI: Magnetic resonance imaging
