# Supplementary figures and images for: Long-term medical imaging use in children with central nervous system tumors
Source: PLoS One. 2021 Apr 21;16(4):e0248643. doi: 10.1371/journal.pone.0248643 (PMC8059842; doi:10.1371/journal.pone.0248643)

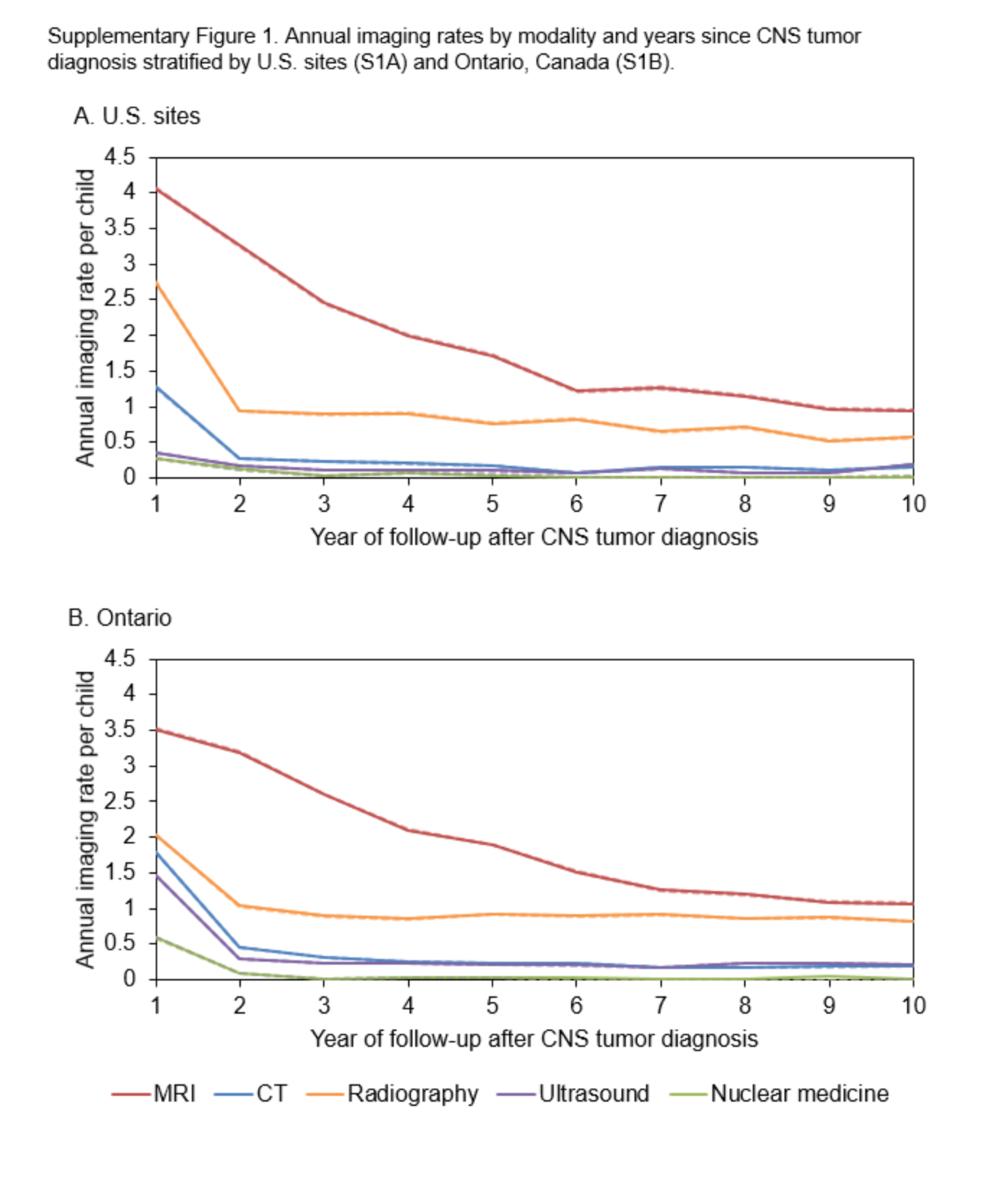

Supplement: S1 Fig — Annual imaging rates by modality and years since CNS diagnosis stratified by U.S. sites (S1A) and Ontario, Canada (S1B). This figure shows the annual imaging rate per child for each imaging modality (MRI [magnetic resonance imaging], CT [computed topography], radiography, ultrasound, and nuclear medicine) for up to 10 years after CNS (central nervous system) tumor diagnosis stratified by U.S. sites (S1A) and Ontario (S1B). The 95% confidence intervals are shown by dashed lines and very closely overlap with the imaging rates. (TIFF) [file pone.0248643.s002.tiff]

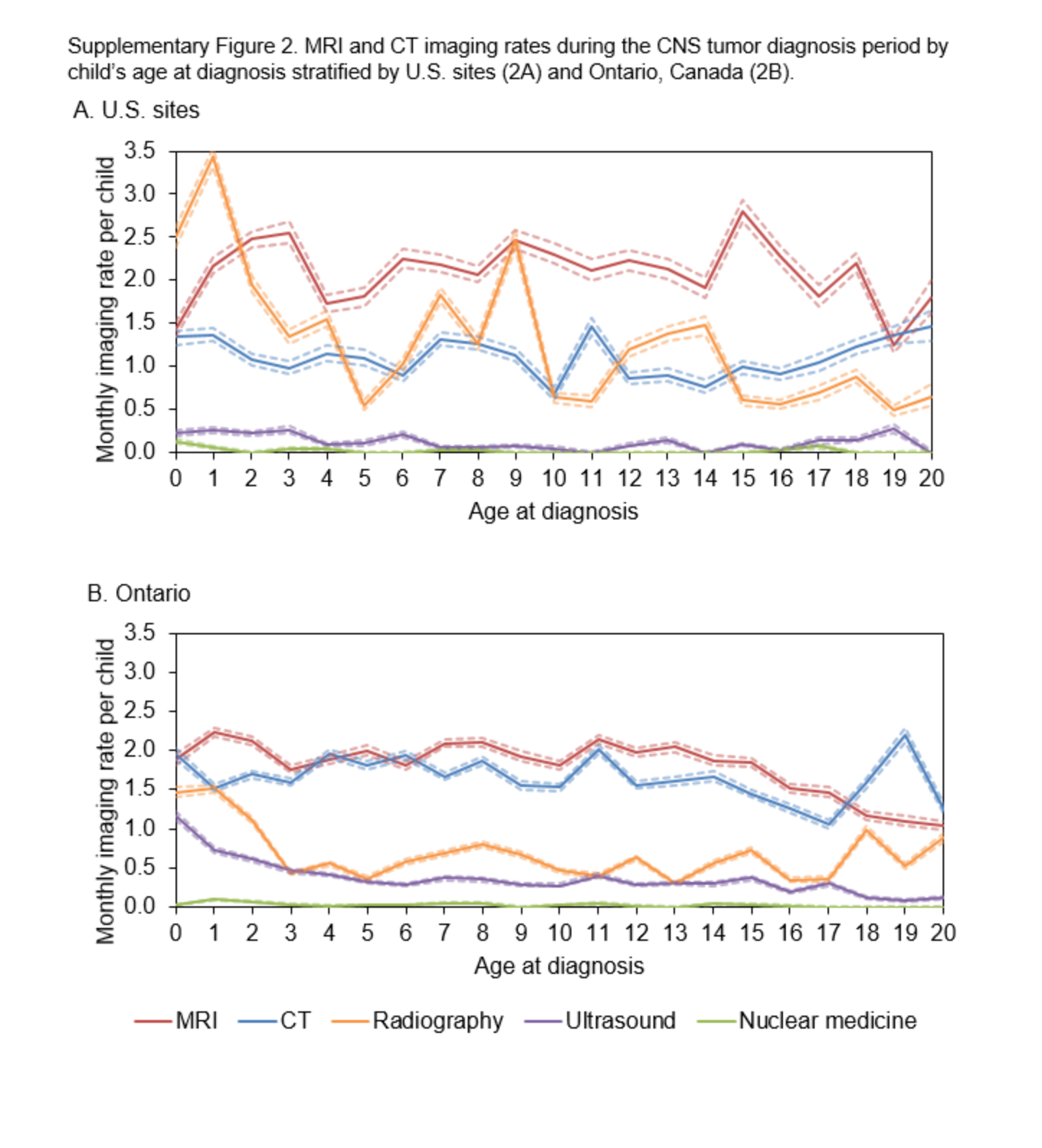

Supplement: S2 Fig — MRI and CT imaging rates during the CNS diagnosis period by child’s age at diagnosis stratified by U.S. sites (2A) and Ontario, Canada (2B). This figure shows the monthly imaging rate per child for each imaging modality (MRI [magnetic resonance imaging], CT [computed topography], radiography, ultrasound, and nuclear medicine) during the CNS (central nervous system) tumor diagnosis period (+/-15 days around the day of diagnosis) stratified by age at diagnosis and by U.S. sites (S2A) and Ontario (S2B). The 95% confidence intervals are shown by dashed lines and very closely overlap with the imaging rates. (TIFF) [file pone.0248643.s003.tiff]

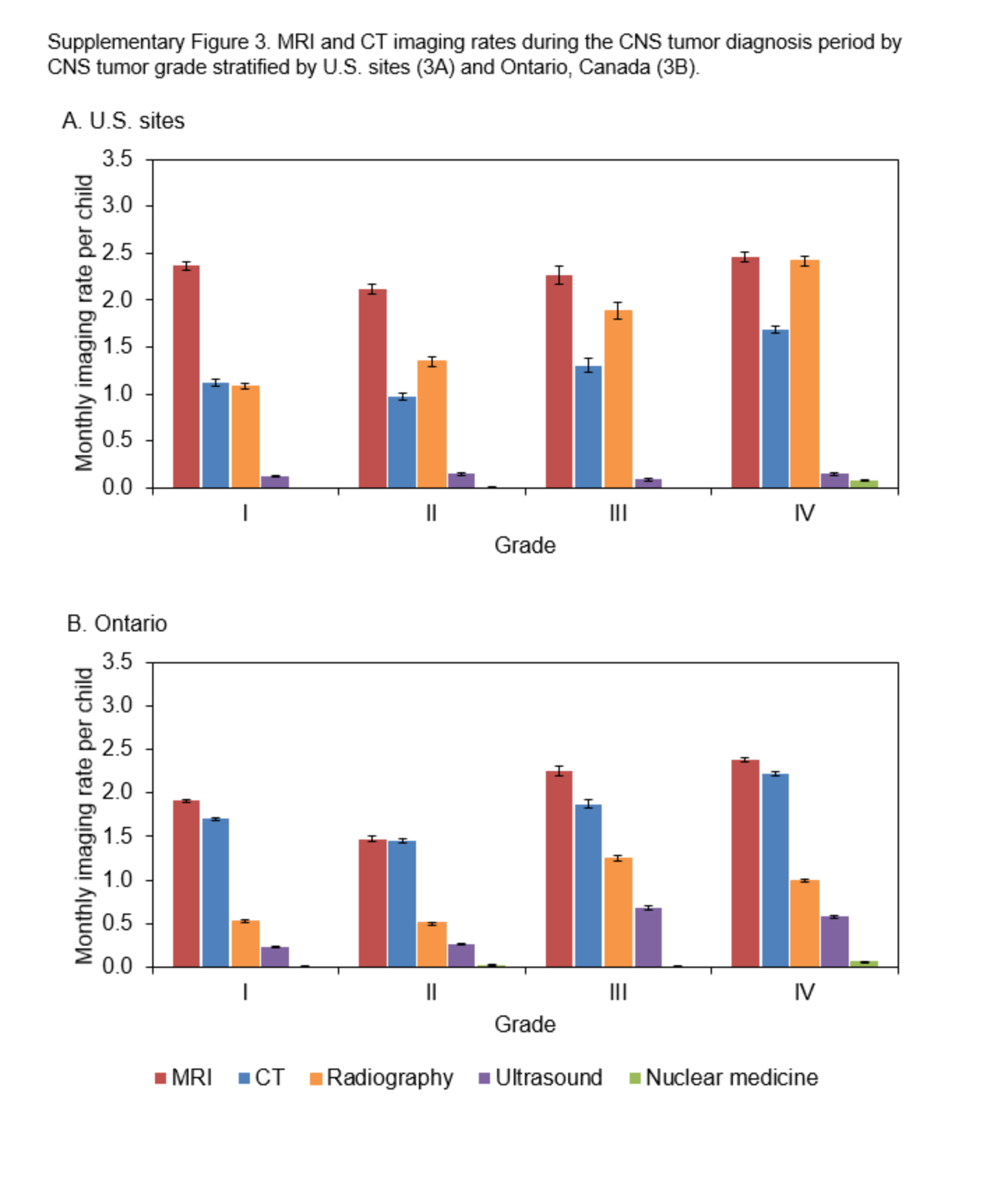

Supplement: S3 Fig — MRI and CT imaging rates during the CNS diagnosis period by CNS tumor grade stratified by U.S. sites (3A) and Ontario, Canada (3B). This figure shows the monthly imaging rate per child for each imaging modality (MRI [magnetic resonance imaging], CT [computed topography], radiography, ultrasound, and nuclear medicine) during the CNS (central nervous system) tumor diagnosis period (+/-15 days around the day of diagnosis) stratified by CNS tumor grade and by U.S. sites (S3A) and Ontario (S3B). The 95% confidence intervals are shown by error bars. (TIFF) [file pone.0248643.s004.tiff]

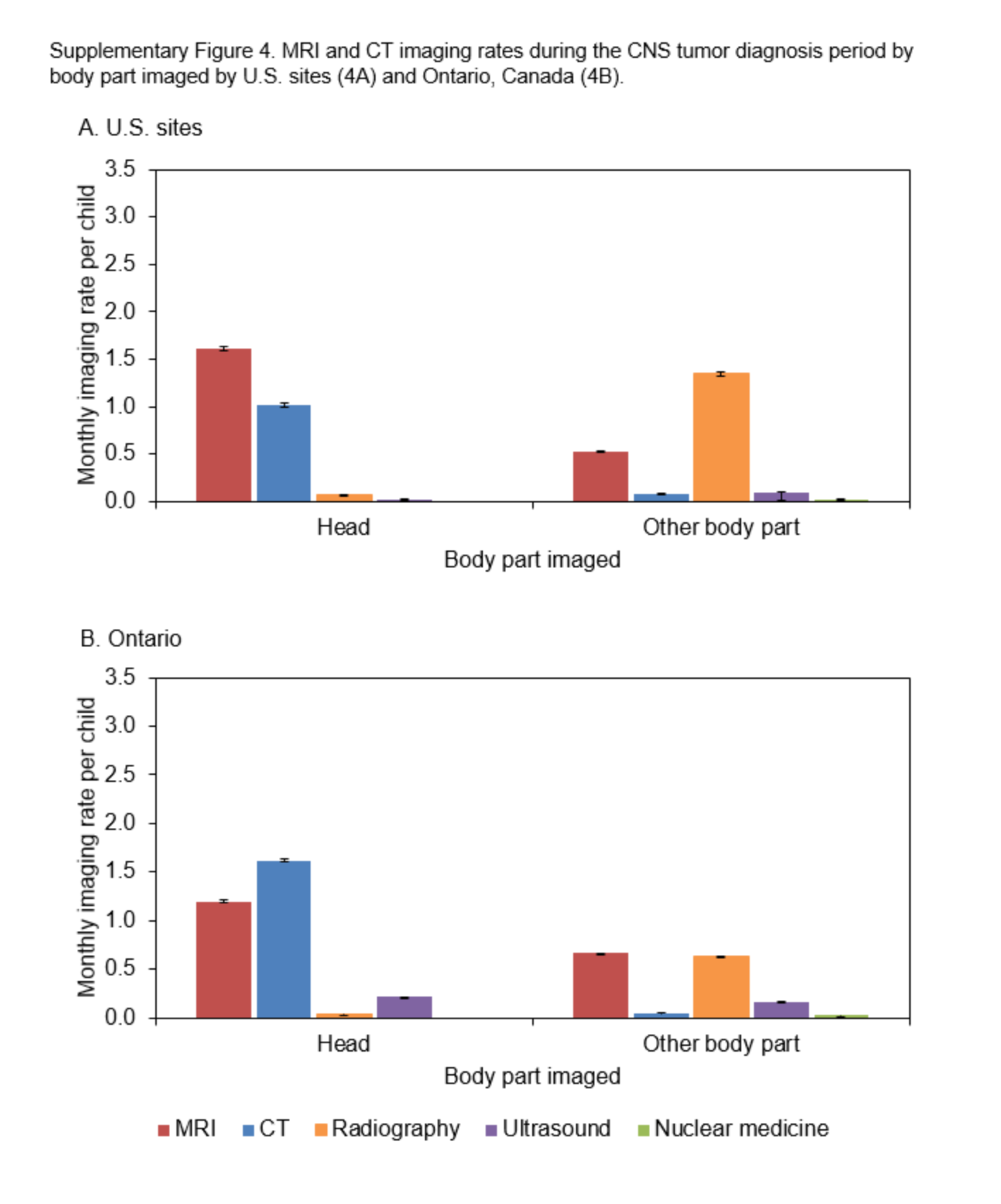

Supplement: S4 Fig — MRI and CT imaging rates during the CNS diagnosis period by body part imaged by U.S. sites (4A) and Ontario, Canada (4B). This figure shows the monthly imaging rate per child for each imaging modality (MRI [magnetic resonance imaging], CT [computed topography], radiography, ultrasound, and nuclear medicine) during the CNS (central nervous system) tumor diagnosis period (+/-15 days around the day of diagnosis) stratified by body part imaged and by U.S. sites (S4A) and Ontario (S4B). The 95% confidence intervals are shown by error bars. (TIFF) [file pone.0248643.s005.tiff]

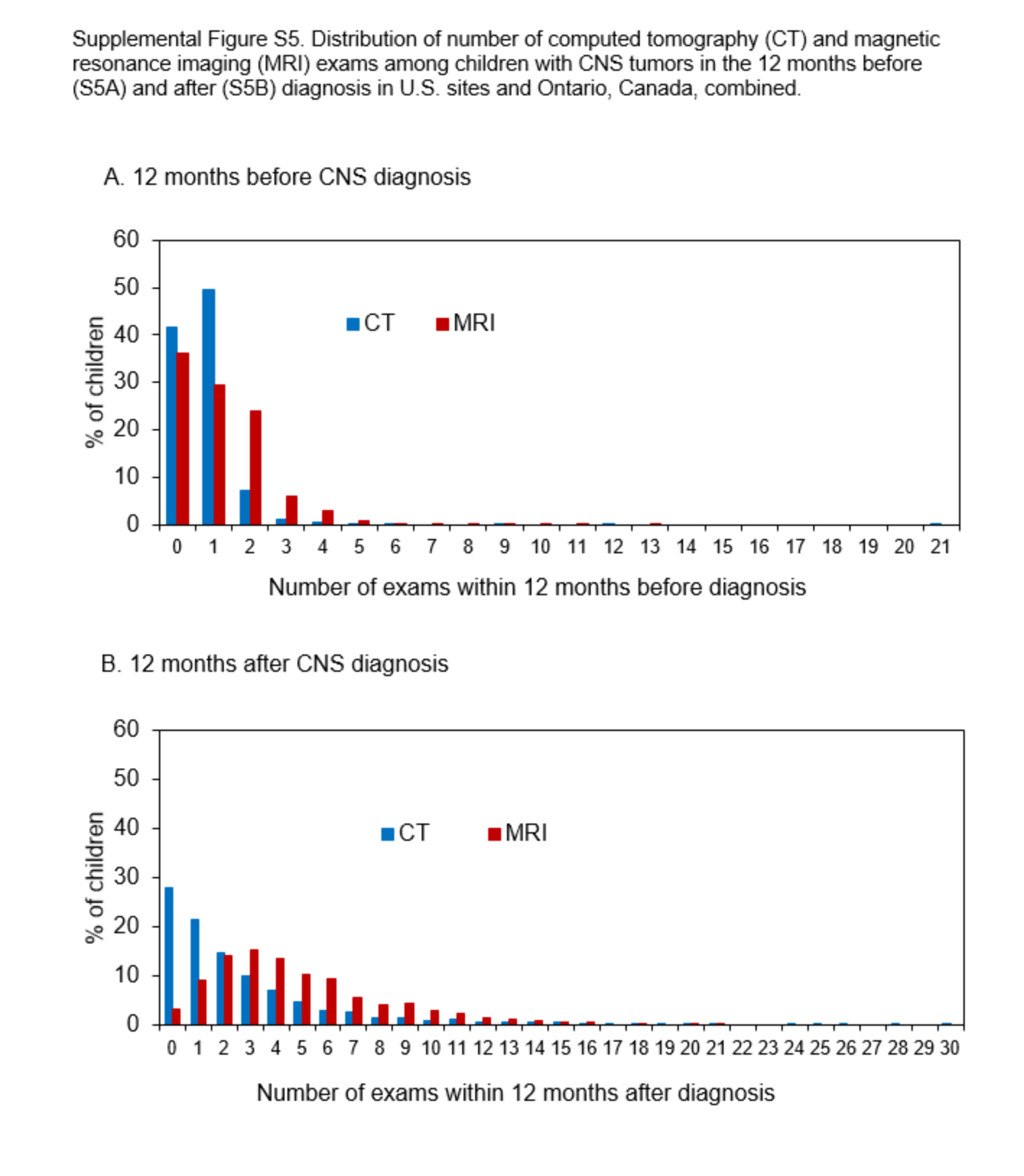

Supplement: S5 Fig — Distribution of number of computed tomography (CT) and magnetic resonance imaging (MRI) exams among children with CNS tumors in the 12 months before (S5A) and after (S5B) diagnosis in U.S. sites and Ontario, Canada, combined. This figure shows the proportion of children by the number of CT (computed topography) and MRI (magnetic resonance imaging) exams they had in the 12 months before CNS (central nervous system) tumor diagnosis (5A) and the 12 months after CNS diagnosis (5B). (TIFF) [file pone.0248643.s006.tiff]
